# Body mass index-associated responses to an ABVD-like regimen in newly-diagnosed patients with Hodgkin lymphoma

**DOI:** 10.3389/fphar.2023.1195907

**Published:** 2023-08-23

**Authors:** Min Hu, Yiduo Ding, Haizhou Zhang, Wei Guo, Yun Li, Zhengming Jin, Changju Qu, Fan Xia

**Affiliations:** ^1^ Department of Pharmacy, The First Affiliated Hospital of Soochow University, Suzhou, China; ^2^ Department of Pharmacy, Union Hospital, Tongji Medical College, Huazhong University of Science and Technology, Wuhan, China; ^3^ Department of Hematology, Collaborative Innovation Center of Hematology, Institute of Blood and Marrow Transplantation, The First Affiliated Hospital of Soochow University, Jiangsu Institute of Hematology, Suzhou, China

**Keywords:** Hodgkin lymphoma, body mass index, ABVD-like regimen, overall response rate, treatment

## Abstract

**Background:** The role of body mass index (BMI) in the treatment outcomes of lymphoma patients is controversial. While investigating the efficacy of ABVD-like regimen in Hodgkin lymphoma (HL) patients, we observed that obese patients had poor responses. To better understand this clinical phenomenon, we evaluated the effect of BMI on responses to ABVD-like chemotherapy in HL patients.

**Methods:** This retrospective cohort study evaluated the clinical outcomes of all 67 patients with confirmed HL who were treated at the First Affiliated Hospital of Soochow University from November 2016 to March 2023 with an ABVD-like regimen as first-line chemotherapy. Baseline patient characteristics and clinical outcomes were compared across different BMI categories. The primary end-point was the overall response rate defined as the proportion of the HL patients who achieved complete response or partial response. The additional end-points included progression-free survival and overall survival.

**Results:** The median age of the HL patients was 31 years old. Of the patients, 10.4% were obese, and 17.9% patients were overweight. Interim and end-term response evaluations revealed overall response rates of 98.5% and 83.6%, respectively. The proportion of patients with potential poor prognostic factors (IPS risk factors) did not differ significantly in the responders versus non-responders. However, non-responders had a higher average BMI when compared with responders (*p* = 0.002). Poor overall response rates in higher BMI patients indeed manifested with shorter progression free survival (*p* = 0.013). The minimum relative dose of the ABVD-like regimen in the overweight and obese groups was significantly lower than in the normal weight group (*p* < 0.001).

**Conclusion:** Our analyses show that >80% of newly-diagnosed HL patients responded to the ABVD-like regimen. We find that being obese or overweight at the time of diagnosis correlated with a poorer overall response rate and that BMI was an independent risk factor in HL patients treated with the ABVD-like regimen. Lower doses of ABVD-like regimen contributed to the discrepant findings of responses in the high BMI groups. These findings indicate that newly-diagnosed, obese HL patients receiving an ABVD-like regimen require personalized treatment.

## 1 Introduction

Hodgkin lymphoma (HL) is characterized by the presence of Reed–Sternberg cells (Shanbhag and Ambinder, 2018). The 2019 Global Burden of Diseases, Injuries, and Risk Factors Study found that China accounts for 10.8% of all new HL cases and 9.8% of HL-associated deaths (Diseases and Injuries, 2020). The standard therapy for HL is the combination of doxorubicin, bleomycin, vinblastine, and dacarbazine (ABVD), which in high-income countries achieves long-term cure rates of >80% (Canellos et al., 2014). However, because of limited drug accessibility, an ABVD-like regimen, in which vindesine or vincristine replaces vinblastine and epirubicin or liposomal doxorubicin replaces doxorubicin, is used in China. Although the ABVD-like regimen is widely used to treat HL patients, patients’ responses are unclear, especially when compared with the standard ABVD regimen. Moreover, different populations exhibit different clinical responses to ABVD-like chemotherapy regimens (Wen et al., 2022). Among them, obese people have implications for chemotherapy administration and dosing (Khouri et al., 2019).

Although HL has excellent outcomes, obese HL patients treated with the ABVD-like regimen at our center have been observed to have poor prognoses and lower overall response rates (ORR) compared with under- or normal weight HL patients. Moreover, obesity and being overweight have emerged as serious public health challenges in China. Data from the China Health and Nutrition Survey revealed that from 1993 to 2015, the age-standardized prevalence of abdominal obesity rose from 12.1% to 26% (Sun et al., 2021). Thus, the impact of body mass index (BMI) on chemotherapy response in HL patients treated with ABVD-like regimens is of high research interest.

However, the impact of BMI on lymphoma outcomes is controversial. Several investigations have found a significant association between high BMI and improved survival in patients with diffuse large B-cell lymphoma, indolent B-cell non-Hodgkin’s lymphoma, and mantle cell lymphoma (Jones et al., 2010; Carson et al., 2012; Weiss et al., 2014; Weiss et al., 2017). High BMI has also been associated with improved overall survival (OS) in Chinese patients with peripheral T-cell lymphoma (Ma et al., 2020). In contrast, other findings indicate that BMI does not affect OS and immune-related adverse events in HL patients treated with the immune checkpoint inhibitor nivolumab (De Filippi et al., 2021). In addition, three prospective phase III clinical trials in the US found that BMI was not significantly associated with the OS and progression-free survival of patients with HL, diffuse large B-cell lymphoma, or follicular lymphoma (Hong et al., 2014), which contradicts previous findings. However, these results are based on survival. Further investigations are needed to determine the importance of the observed discrepancies. Considerable effort is being put into identifying the tumor and host factors that determine ORR following treatment with ABVD-like regimens, which may significantly improve the first-line therapy landscape of HL in China. Until now, there is no clear evidence of a role of baseline BMI in Chinese HL patients.

In this retrospective study involving newly-diagnosed Chinese HL patients, we assessed the efficacy of an ABVD-like regimen and examined the potential impact of BMI on clinical outcomes following the use of the ABVD-like regimen as first-line therapy. The study’s main objective is to recommend guidelines for the personalized treatment of obese HL patients in China.

## 2 Materials and methods

### 2.1 Patients and treatment

Ethics approval for the study was granted by the ethics committee of the institution where the study was conducted by obtaining the study subjects’ consent. The study adhered to the Declaration of Helsinki guidelines. The study involved newly-diagnosed HL patients who were treated at the First Affiliated Hospital of Soochow University using an ABVD-like regimen as first-line therapy between November 2016 and March 2023. Individual patient records were correlated with clinical information, including weight and height. Any missing data were obtained from electronic medical records when available. A total of 149 HL patients were identified and 67 newly-diagnosed patients, treated with an ABVD-like regimen, were included in this study. Relapsed/refractory patients or those treated with other first-line therapies were excluded from the analysis. The ABVD-like regimen was made of dacarbazine (375 mg/m^2^), vindesine (fixed dosage, 4 mg), bleomycin (10 mg/m^2^), and doxorubicin (25 mg/m^2^)/epirubicin (30–40 mg/m^2^)/liposomal doxorubicin (20–30 mg/m^2^) on days 1 and 15 of each cycle (4 weeks per cycle). For some patients, the dosage of the ABVD-like regimen was decreased or its administration timing was postponed based on the attending physician’s judgment.

### 2.2 BMI measurements and data collection

BMI was calculated using the formula, weight (kg)/height (m)^2^ based on the data collected at patient diagnosis. Based on the World Health Organization (WHO) guidelines, BMIs of <18.5 kg/m^2^, 18.5 kg/m^2^ to <25 kg/m^2^, 25 kg/m^2^ to <30 kg/m^2^, and ≥30 kg/m^2^, indicated underweight, normal weight, overweight, and obesity, respectively (Kivimäki et al., 2022). Using 25 kg/m^2^ as the cutoff, patients were categorized into the low BMI group or the high BMI group. Clinical data, including sex, age, BMI, histologic subtype, Ann Arbor stage, B symptoms, the Eastern Cooperative Oncology Group performance score, IPS (International Prognostic Score) risk factors, blood parameters, treatment regimens, and drug dosage, were recorded at the time of diagnosis (De Filippi et al., 2021).

### 2.3 Treatment response

The study’s primary end-point was to determine the role of BMI in clinical response by HL patients treated with ABVD-like chemotherapy. Treatment responses were assessed using computed tomography (CT) scanning with contrast and/or fluorodeoxyglucose positron emission tomography/computed tomography (PET/CT). Complete response (CR), partial response (PR), stable disease (SD), and progressive disease (PD) were estimated after the ABVD-like treatment using the Lugano criteria (Cheson et al., 2014). Interim response and end-term response evaluations were done 2 weeks after cycle 2 by contrast CT or PET/CT and 6 weeks after cycle 6 by PET/CT, respectively. The primary end-point was the ORR, defined as the proportion of patients who achieved CR or PR. To identify risk factors that affect response to the ABVD-like regimen, patients with CR or PR were categorized as the responders, whereas those with SD or PD were categorized as the non-responders. The additional end-points included assessment of progression-free survival (PFS) and overall survival (OS).

### 2.4 Statistical analyses

Statistical analyses were done on SPSS version 20.0. Categorical variables are presented as frequencies and percentages. Continuous variables were analyzed using descriptive statistical analysis (median and range) and the Mann-Whitney *U* test. Where indicated, clinical factors were compared using an independent *t*-test, Fisher’s exact test, Pearson’s Chi-square test, and the Mann-Whitney *U* test. Multivariable analysis was done using the Cox regression model. In the Cox models, data were expressed as relative risk (RR) and 95% confidence intervals (CI). PFS and OS analyses were performed using the Kaplan–Meier method. *p* ≤ 0.05 indicated statistically significant differences.

## 3 Results

### 3.1 Patients’ characteristics

The mean age of the 67 patients included in the study was 31 years (range: 13–72 years). Thirty-eight (56.7%) of the patients were male. The median BMI was 23.6 kg/m^2^ (range: 17.2–38.5 kg/m^2^). Based on the WHO criteria, seven patients (10.4%) were underweight, 41 (61.2%) had normal weight, 12 (17.9%) were overweight, and seven (10.4%) were obese. The most prevalent histologic HL subtype was nodular sclerosis (58.2%). Thirty-one (46.3%) patients had stage Ⅲ–Ⅳ HL, 17 (25.4%) had B symptoms, and 21 (31.3%) had extranodal involvement. Patients had good Eastern Cooperative Oncology Group performance status (0–1 in 97%). Only a minority of patients presented IPS risk factors, which are associated with poorer prognoses in other settings (Hasenclever and Diehl, 1998). Detailed clinical characteristics are shown in Table 1.

**TABLE 1:** Demographic and clinical characteristics of the 67 HL patients.

Characteristics (*n* = 67)	No. (%)
Age, years (Median, range)	31 (13–72)
BMI, kg/m^2^ (Mean, range)	23.6 (17.2–38.5)
Underweight (BMI <18.5)	7 (10.4)
Normal weight (18.5 ≤ BMI <25)	41 (61.2)
Overweight (25 ≤ BMI <30)	12 (17.9)
Obese (BMI ≥30)	7 (10.4)
Histologic subtype (*n*, %)	
Classic HL, mixed cellularity	16 (23.9)
Classic HL, nodular sclerosis	39 (58.2)
Classic HL, lymphocyte rich	11 (16.4)
Nodular lymphocyte predominant	1 (1.5)
Ann Arbor stage (*n*, %)	
Ⅰ–Ⅱ	36 (53.7)
Ⅲ–Ⅳ	31 (46.3)
B symptoms	17 (25.4)
ECOG performance status (*n*, %)	
0–1	65 (97.0)
≥2	2 (3.0)
Extranodal involvement (*n*, %)	21 (31.3)
Blood parameters at diagnosis (Mean, range)	
Serum albumin level, g/L	41.2 (25.2–51.5)
Hemoglobin level, g/L	128 (85–170)
WBC count, 10^9^/L	8.86 (2.13–21.38)
Lymphocyte count, 10^9^/L	1.40 (0.24–3.28)
IPS risk factors (n, %)	
Sex-Male (*n*, %)	38 (56.7)
Age ≥45 years	21 (31.3)
Ann Arbor stage IV	17 (25.4)
Serum albumin <40 g/L	25 (37.3)
Hemoglobin level <105 g/L	6 (9.0)
Lymphocyte count <0.6 × 10^9^/L	6 (9.0)
WBC count ≥15 × 10^9^/L	6 (9.0)

BMI, body mass index; ECOG, eastern cooperative oncology group; HL, hodgkin lymphoma; IPS, international prognostic score; WBC, white blood cell.

### 3.2 Treatment and clinical efficacy

**FIGURE 1:**
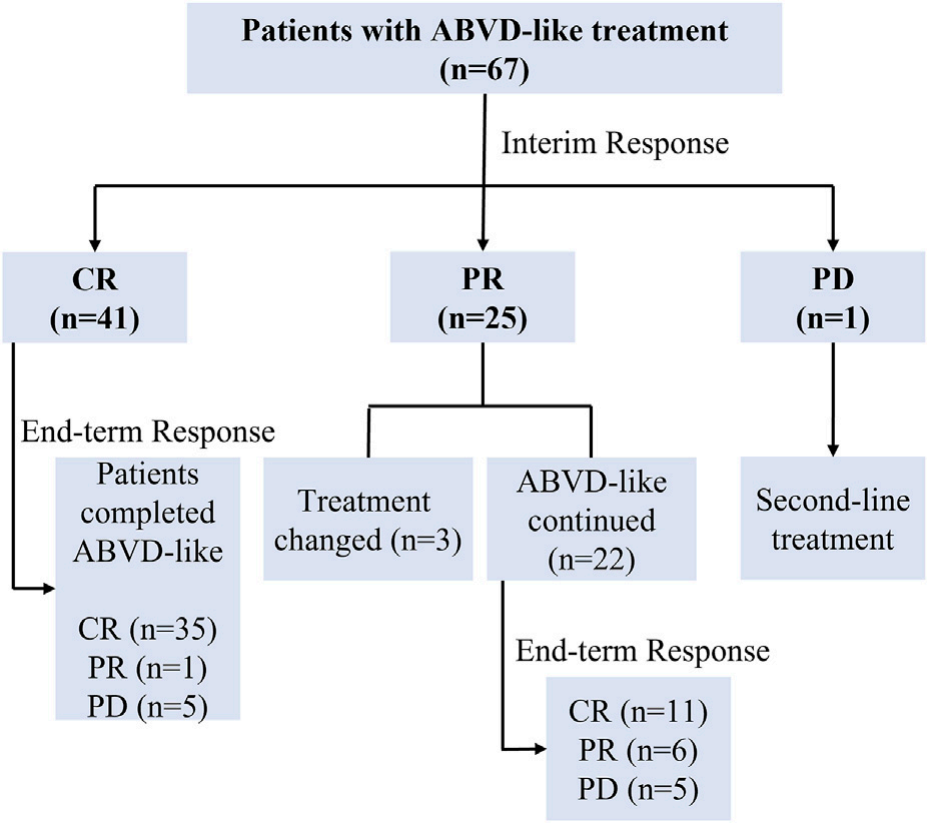
Flow chart of patients’ treatments. ABVD-like, doxorubicin, bleomycin, vindesine, and dacarbazine-like; CR, complete response; PR, partial response; PD, progressive disease.

Figure 1 shows a flow chart of patients’ treatment strategies. Table 2 shows the interim and end-term responses to the ABVD-like regimen. The interim response evaluation showed that 41 patients (61.2%) had CR, 25 (37.3%) had PR, and one (1.5%) had PD, indicating an ORR of 98.5%. Of the 25 patients with PR, three changed the subsequent treatment while the remaining 22 patients stayed on the ABVD-like treatment. The 41 patients with CR maintained the previous ABVD-like treatment. The patient with PD received second-line treatment. End-term response analysis showed that 46 patients (68.7%) achieved CR while 10 patients (14.9%) achieved PR, indicating an ORR of 83.6%. The remaining 11 patients (16.4%) did not respond to the ABVD-like treatment. Overall, at our center, the ABVD-like chemotherapy was associated with an ORR of >80%. Furthermore, at 48 months of median follow-up (range: 17-80), all patients were alive with 100% OS rate. The 2-year, 3-year and 5-year PFS rates were 65.7%, 58.2% and 29.9% respectively, which was independent of whether or not that patient received radiotherapy (Supplementary Table S1).

**TABLE 2:** Analysis of interim and end-term responses to the ABVD-like regimen.

Response to ABVD-like treatment	Interim response		End-term response	
	No. of patients	%	No. of patients	%
CR	41	61.2	46	68.7
PR	25	37.3	10	14.9
SD	0	0	0	0
PD	1	1.5	11	16.4
ORR (%)	98.5		83.6	

Abbreviations: CR, complete response; PR, partial response; SD, stable disease; PD, progressive disease.

### 3.3 Comprehensive characterization of responders and non-responders

To address concerns about the quality of the best response, comparisons were made between responders and non-responders. As shown in Table 3, the end-term response to the ABVD-like regimen was not associated with the disease-associated baseline features determined at diagnosis, such as age, sex, stage Ⅲ–Ⅳ, B symptoms, extranodal involvement, serum albumin level, hemoglobin level, and lymphocyte count. The responder and non-responder groups did not differ significantly with regard to the proportion of patients with potential poor prognostic factors (IPS risk factors). Specifically, non-responders had a higher average BMI than responders (*p* = 0.002). The non-responder group had significantly a smaller proportion of patients with normal weight (*p* = 0.029) and a significantly higher proportion of obese patients (*p* = 0.011). Additionally, at diagnosis, the average white blood cell count was significantly higher in non-responders (*p* = 0.028). Further, we also analyzed clinical variables in metabolic CR (mCR) vs. no-mCR. As is shown in Supplementary Table S2, the patients charateristics between two subgroups were comparable and no-mCR group had more obese patients than mCR group (*p* = 0.047) which was consistent with the result in responders vs. non-responders.

**TABLE 3:** Patients’ characteristics before ABVD-like treatment.

Characteristics	Responders (*n* = 56)	Non-responders (*n* = 11)	*p*-value
Age, years (Median, range)	31 (17–72)	24 (13–67)	0.472
BMI (Mean, range)	22.8 (17.2–38.5)	27.8 (18.5–38.4)	0.002
Underweight (*n*, %)	7 (12.5)	0 (0)	0.589
Normal weight (*n*, %)	38 (67.9)	3 (27.3)	0.029
Overweight (*n*, %)	8 (14.3)	4 (36.4)	0.188
Obese (*n*, %)	3 (5.4)	4 (36.4)	0.011
Ann Arbor stage Ⅲ–Ⅳ (*n*, %)	26 (46.4)	5 (45.5)	0.953
B symptoms (*n*, %)	15 (26.8)	2 (18.2)	0.825
Extranodal involvement (*n*, %)	18 (32.1)	3 (27.3)	0.537
Blood parameters at diagnosis (Mean, range)			
Serum albumin level, g/L	41.2 (27.8–51.5)	41.0 (25.2–47.3)	0.916
Hemoglobin level, g/L	127 (85–170)	132 (96–157)	0.464
WBC count, 10^9^/L	8.20 (2.13–16.57)	12.21 (3.72–21.38)	0.028
Lymphocyte count, 10^9^/L	1.38 (0.24–3.28)	1.52 (10.87–2.35)	0.510
IPS risk factors (*n*, %)			
Sex-Male (*n*, %)	30 (53.6)	8 (72.7)	0.241
Age ≥45 years	18 (32.1)	3 (27.3)	1.000
Ann Arbor stage IV	14 (25.0)	3 (27.3)	1.000
Serum albumin <40 g/L	23 (41.1)	2 (18.2)	0.274
Hemoglobin level <105 g/L	5 (8.9)	1 (9.1)	1.000
WBC count ≥15 × 10^9^/L	3 (5.4)	3 (27.3)	0.080
Lymphocyte count <0.6 × 10^9^/L	6 (10.7)	0 (0)	0.579
Anthracycline chemotherapy drugs			
Doxorubicin/epirubicin	20 (35.7)	5 (45.5)	0.541
Doxorubicin hydrochloride liposome	36 (64.3)	6 (54.5)	

Abbreviations: BMI, body mass index; IPS, international prognostic score; WBC, white blood cell.

### 3.4 Efficacy and survival analysis based on BMI

**FIGURE 2:**
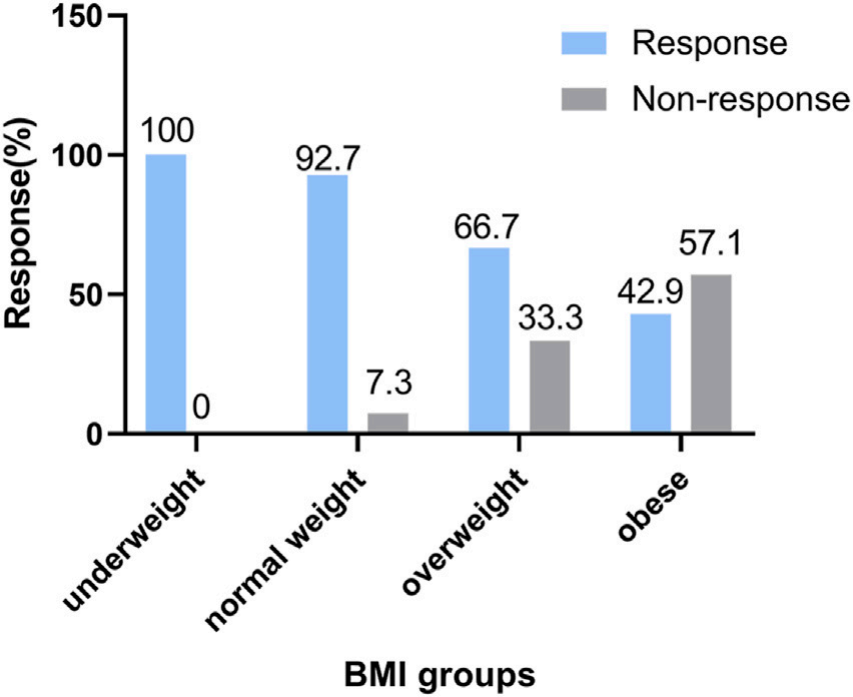
Analysis of treatment efficacies across BMI groups. Association between response and body mass index categories (based on WHO’s criteria) in HL patients treated with the ABVD-like regimen. The response rate is expressed as a percentage.

**FIGURE 3:**
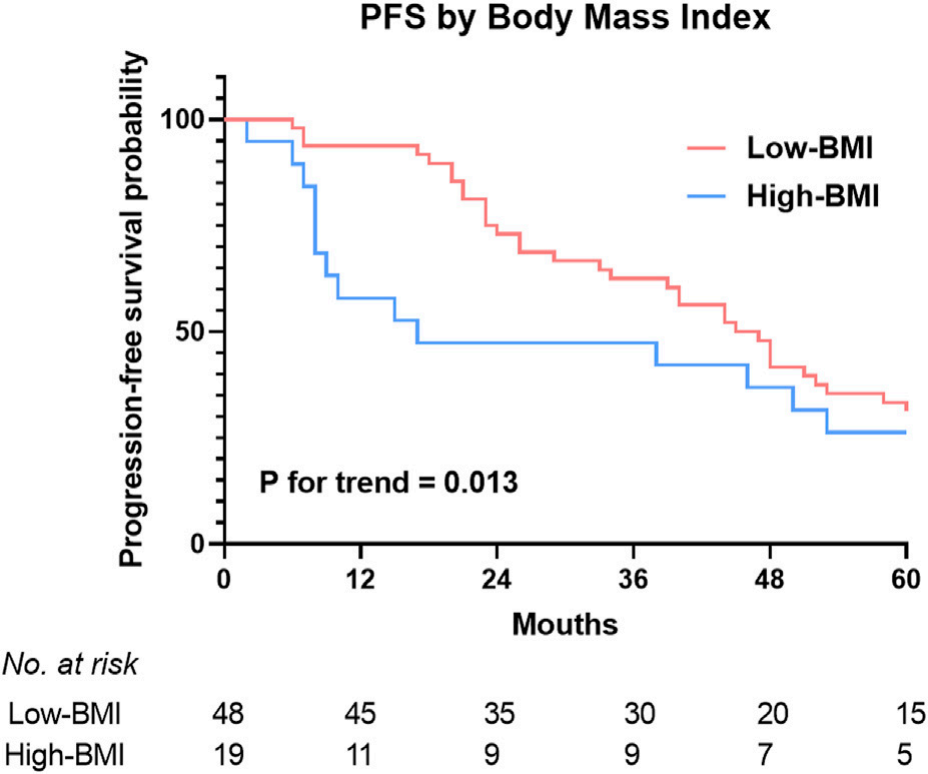
5-year rates of progression free survival estimates in HL patients treated with the ABVD-like regimen according to BMI categories. Landmark analysis of PFS by best response and BMI. PFS, progression free survival.

Figure 2 shows the BMIs (based on the WHO criteria) of the responders and non-responders. The ORR was 100% in the underweight group, 92.7% in the normal weight group, 66.7% in the overweight group, and 42.9% in the obese group. Higher BMI was associated with poorer prognosis in newly-diagnosed HL patients treated with the ABVD-like regimen. Being obese at diagnosis was associated with lower ORR. In addition, there was significant difference in 5-year PFS rates between low BMI and high BMI groups (*p* = 0.013, Figure 3). 2-year, 3-year, and 5-year PFS rates for patients with high BMI were 47.4%, 47.4%, and 26.3% respectively (Table 4).

**TABLE 4:** 2-*year*, 3-*year,* and 5-*year* rates of progression free survival between low BMI and high BMI groups.

BMI group	2-*year*		3-*year*		5-*year*	
	No. patients	%	No. patients	%	No. patients	%
low BMI (*n* = 48)	35	72.9	30	62.5	15	31.3
high BMI (*n* = 19)	9	47.4	9	47.4	5	26.3

Abbreviations: BMI, body mass index.

### 3.5 BMI as an independent predictor of ORR

**FIGURE 4:**
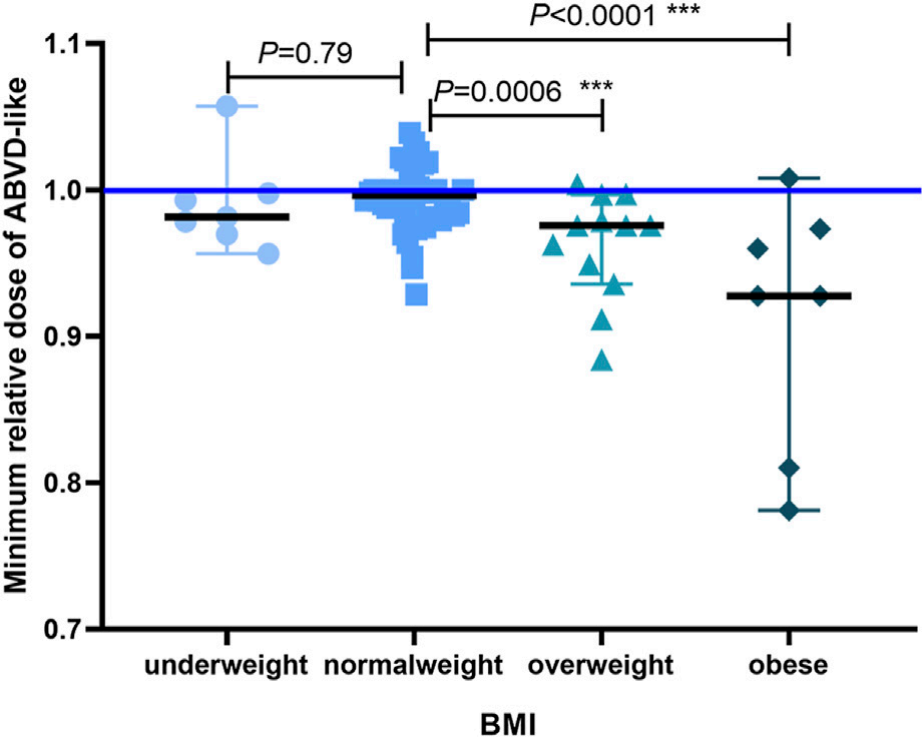
The minimum relative dose of the administered ABVD-like regimen. We defaulted vindesine, bleomycin, and doxorubicin/epirubicin/liposomal doxorubicin to relative number 1, and the minimum relative dose of ABVD-like regimen converted with dacarbazine was shown as median with 95% CI (***p < 0.001).

To assess the prognostic value of BMI when compared with established prognostic markers, we carried out an IPS risk factor analysis in the low and high BMI groups. Univariate analysis revealed that separately, IPS risk factors like sex, age, Ann Arbor stage IV, serum albumin levels, hemoglobin levels, WBC count, and lymphocyte count did not differ significantly between the low and high BMI groups (Table 5). Next, significant factors were included in a multivariate Cox regression model. The multivariate analysis only revealed significant differences about high BMI (RR = 6.737, *p* = 0.02, Table 6). Obesity has implications for chemotherapy administration and dosing (Matos et al., 2016). Next, we analyzed the impact of obesity on ORR as well as the key outcomes that were associated with dosage in the HL patients treated with the ABVD-like regimen (Figure 4). For most cancers, chemotherapy dosing is based on estimated body surface area. Our analysis based on the four BMI groups (using the WHO criteria), showed that the minimum relative dose of the ABVD-like regimen in the overweight and obese groups was significantly lower than in the normal weight group (*p* < 0.001).

**TABLE 5:** IPS risk factors in the low versus high BMI groups.

IPS risk factor	Low BMI (*n* = 48)		High BMI (*n* = 19)		*p*-value
	No. patients	%	No. patients	%	
Sex-Male	25	52.1	13	68.4	0.224
Age ≥45 years	13	27.1	8	42.1	0.232
Ann Arbor stage IV	15	31.3	2	10.5	0.148
Serum albumin <40 g/L	20	41.7	5	26.3	0.242
Hemoglobin level <105 g/L	5	10.4	1	5.3	0.848
WBC count ≥15 × 10^9^/L	3	6.3	3	15.8	0.448
Lymphocyte count <0.6 × 10^9^/L	6	12.5	0	0	0.173

Abbreviations: BMI, body mass index; IPS, international prognostic score; WBC, white blood cell.

**TABLE 6:** Multivariate analyses of clinical variables in relation to response to ABVD-like treatment.

Factor	RR	95% CI	*p*-Value
High BMI	6.737	1.997–22.726	0.020
IPS risk factors			
Sex-Male (*n*, %)	2.035	0.591–7.003	0.587
Age ≥45 years	0.881	0.260–2.983	0.945
Ann Arbor stage IV	1.103	0.330–3.689	0.234
Serum albumin <40 g/L	0.373	0.088–1.592	0.130
Hemoglobin level <105 g/L	1.017	0.156–6.641	0.957
WBC count ≥15 × 10^9^/L	3.813	1.363–10.661	0.701
Lymphocyte count <0.6 × 10^9^/L	1.220	1.085–1.372	0.999

Abbreviations: RR, relative risk; BMI, body mass index; IPS, international prognostic score; WBC, white blood cell.

## 4 Discussion

Although HL is highly curable in high-income countries, many patients worldwide do not have access to the standard ABVD regimen. ABVD-like regimens have recently emerged as first-line chemotherapy in China (Wen et al., 2022). In this study, we report the use of the combination of dacarbazine, vindesine, bleomycin, and doxorubicin/epirubicin/liposomal doxorubicin on days 1 and 15 of each cycle as first-line therapy for patients with newly-diagnosed HL. Interim and end-term response analyses revealed ORRs of 98.5% and 83.6%, respectively. Overall, the ABVD-like regimen was associated with an ORR of >80%. Moreover, a significant association was observed between high BMI (≥25 kg/m^2^) and poorer ORR in HL patients treated with the ABVD-like regimen, even after adjusting for other clinical factors. To the best of our knowledge, most studies have focused on the effect of BMI on survival and the effect of BMI on ORR in HL has not been explored. According to the present study, we explored the effect of BMI on ORR considering that, on one hand, HL patients usually had a long survival including in those relapsed/refractory HL patients with the development of new drugs like programmed cell death protein 1 (PD-1) inhibitors, CD30 inhibitors; on the other hand, ORR may translate to improved survival with a long follow-up period (Ma et al., 2021). In our study, poor ORR in higher BMI patients indeed manifested with shorter PFS (*p* = 0.013).

In this study’s cohort of Chinese patients with newly-diagnosed HL, being overweight or obese at the time of diagnosis was associated with low ORR, which is inconsistent with previous reports (Hong et al., 2014; De Filippi et al., 2021). This discrepancy may be caused by the following reasons. First, the studies involve different racial and ethnic groups. Second, the cause of the discrepancy may be multi-fold, involving interaction between a specific drug type, dose, and pharmacokinetics (Matos et al., 2016; Wen et al., 2022). Third, differences in the level of therapeutic exposure in obese versus normal weight individuals might confound the observed effects (Aminian et al., 2022). Additionally, unlike our study, which examined the ORR and PFS of a first-line treatment, previous studies examined survival, which may be affected by different salvage treatments. Further investigations are needed to understand the reasons for the discrepancies between studies, as well as to characterize the effects of BMI on treatment and response.

To investigate the risk factors contributing to poor responses in the high BMI groups, we compared the IPS risk factors among different BMI categories and found that the proportion of patients with different IPS risk factors was comparable. Moreover, IPS risk factors did not differ significantly between responders and non-responders. Lower doses of ABVD-like regimen can contribute to the differences in the observations made across BMI groups. As is well known, the improved outcomes are achieved with standard chemotherapy regimens in a dose-dependent manner and patients receiving higher dose intensities experience better survival (Denduluri et al., 2015; Havrilesky et al., 2015). However, the patients with higher BMI may have altered pharmacokinetics and potential for differences in drug distribution, which lead to increased drug exposure and risk of treatment-related toxicity (Navarro, 2003; Morrish et al., 2011). Consequently, some practitioners may reduce chemotherapy doses empirically in this patient population. Our study showed that patients with a higher BMI were treated at relatively low dose intensities, representing a potential reason for lower ORR and shorter PFS. Our results confirmed once again the importance of “full weight-based dosing” from ASCO guidelines (Griggs et al., 2021).

Dose individualization is a viable strategy that can improve outcomes in obese patients. If obese HL patients tolerate full uncapped doses of chemotherapy with no increase in toxicity, and a similar response to that seen in non-obese patients. Our study would provide recommendations for the individualized treatment strategies in obese patients with newly diagnosed HL treated with ABVD-like regimen. However, due to small sample size and retrospective analysis in this study, large-scale randomized controlled phase III trial should be guaranteed in the future to figure out the effect of BMI on response to ABVD-like regimen in HL patients.

## 5 Conclusion

Here, we find that newly-diagnosed Chinese HL patients had a response rate of >80% to the ABVD-like regimen. Being overweight or obese at the time of HL diagnosis was associated with lower ORR and BMI was an independent risk factor in HL patients treated with the ABVD-like regimen as first-line treatment. We find that low doses of ABVD-like regimen contribute to the lower response in the high BMI groups. These findings suggest that newly-diagnosed obese HL patients should receive personalized ABVD-like regimens.

## Data Availability

The original contributions presented in the study are included in the article/Supplementary Material, further inquiries can be directed to the corresponding authors.
