# Supplementary material for: Body mass index-associated responses to an ABVD-like regimen in newly-diagnosed patients with Hodgkin lymphoma
Source: Front Pharmacol. 2023 Aug 23;14:1195907. doi: 10.3389/fphar.2023.1195907 (PMC10482088; doi:10.3389/fphar.2023.1195907)
Supplement: Supplementary file 1 [file DataSheet1.pdf]

Supplementary Material

Body Mass Index-Associated Responses to an ABVD-Like Regimen in Newly-Diagnosed Patients with Hodgkin Lymphoma

Min Hu[1,2,†], Yiduo Ding[1,†], Haizhou Zhang[1, †], Wei Guo[2], Yun Li[1], Zhengming Jin[3], Changju Qu[3,*], Fan Xia[1,*]

[†]These authors have contributed equally to this work and share first authorship.

***Correspondence:**
Fan Xia
sabvsmass@sina.com
Changju Qu
qcj310@suda.edu.cn

1 Supplementary Table

Supplemental Table 1. 2-*year*, 3-*year* and 5-*year* rates of progression free survival between radiotherapy group and no-radiotherapy group.

	2-*year*		3-*year*		5-*year*	
	No. patients	%	No. patients	%	No. patients	%
Radiotherapy (n = 13)	11	84.6	8	61.5	3	23.1
No-radiotherapy (n = 54)	33	61.1	31	57.4	17	31.5
Total (n = 67)	44	65.7	39	58.2	20	29.9
P value	0.202		0.786		0.797	

Supplementary Table 2. Association between quality of response and clinical variables in patients with newly diagnosed Hodgkin lymphoma treated with ABVD-like regimen.

**Characteristics**	**mCR (n=46)**	**no-mCR (n=21)**	***P* value**
**Age, years (Median, range)**	31 (23-49)	26 (21-50)	0.354
**BMI (Mean, range)**	22.5 (19.6-24.7)	23.4 (19.2-29.5)	0.242
underweight (n, %)	6 (13.0)	1 (4.8)	0.550
normal weight (n, %)	30 (65.2)	11 (52.4)	0.317
overweight (n, %)	8 (17.4)	4 (19.0)	1.000
obese (n, %)	2 (4.3)	5 (23.8)	0.047
**Ann Arbor stage III-IV (n, %)**	18 (39.1)	13 (61.9)	0.083
**B symptoms (n, %)**	14 (30.4)	3 (14.4)	0.268
**Extranodal involvement (n, %)**	14 (30.4)	7 (33.3)	0.812

**Blood parameters at diagnosis (Mean, range)**			
Serum albumin level, g/L	41.8 (30.9-51.5)	39.8 (25.2-48.7)	0.156
Hemoglobin level, g/L	128 (85-170)	129 (91-157)	0.820
WBC count, 10^9/L	8.39 (2.13-16.57)	9.89 (3.72-21.38)	0.181
Lymphocyte count, 10^9/L	1.43 (0.24-3.28)	1.34 (0.47-2.35)	0.578
**IPS risk factors (n, %)**			
Sex-Male (n, %)	26 (56.5)	12 (57.1)	0.962
Age ≥ 45 years	15 (32.6)	6 (28.6)	0.741
Ann Arbor stage IV	10 (21.7)	7 (33.3)	0.312
Serum albumin < 40 g/L	17 (37.0)	8 (38.1)	0.929
Hemoglobin level < 105 g/L	5 (10.9)	1 (4.8)	0.726
WBC count ≥ 15×10^9/L	3 (6.5)	3 (14.3)	0.568
Lymphocyte count < 0.6×10^9/L	4 (8.7)	2 (9.5)	1.000
**Anthracycline chemotherapy drugs**			0.317
Doxorubicin/epirubicin	19 (41.3)	6 (28.6)	
Doxorubicin hydrochloride liposome	27 (58.7)	15 (71.4)	
